# CGT 4.0: a distant dream or inevitable future? Smart process automation is critical to make efficient scalability of CGT manufacturing a reality 

**DOI:** 10.3389/fbioe.2025.1563878

**Published:** 2025-03-19

**Authors:** Aleksander Szarzynski, Oliver Spadiut, Matthias Reisbeck, Gerhard Jobst, Rachel L. Paterson, Anna Kamenskaya, Emilie Gateau, Hanna P. Lesch, Luc Henry, Bence Kozma

**Affiliations:** ^1^ Research Unit of Biochemical Engineering, Institute of Chemical, Environmental and Bioscience Engineering, TU Wien, Vienna, Austria; ^2^ Jobst Technologies GmbH, Freiburg, Germany; ^3^ Stemmatters Biotecnologia e Medicina Regenerativa SA, Guimarães, Portugal; ^4^ Da Vinci Labs SAS, Tours, France; ^5^ Exothera SA, Jumet, Belgium; ^6^ Limula SA, Épalinges, Switzerland

**Keywords:** cell and gene therapy, automation, sensors, PAT, single-use, digital twin, manufacturing, viral vector

## 1 Introduction

Cell and gene therapies (CGTs) are new treatment modalities with demonstrated clinical results against a wide range of hard-to-treat diseases (Kliegman et al., 2024; Kohn et al., 2023). They are either *ex vivo* treatments, obtained by manipulating cells in a laboratory before returning them to the patient, or *in vivo* applications, that involve direct injection of genetic material into the bloodstream or a target organ (Sainatham et al., 2024). Cell and gene therapy (CGT) products are significantly different from previous generations of biologics, such as recombinant proteins or vaccines, and have been challenging the production capabilities, supply chain and business models of the pharmaceutical industry (Tarnowski et al., 2017; Shah et al., 2023). With the CGT market still in its infancy, even a decade after the first market approvals, three factors related to their manufacturing have been limiting the broad adoption of CGT products in the clinic: 1) the highly variable starting materials due to the personalised nature of the treatments (Heathman et al., 2016; Cuffel et al., 2022), 2) the diversity and complexity of their production processes (Lowdell, 2024), and 3) the lack of fit-for-purpose tools supporting scalable supply at the commercial stage (Garcia-Aponte et al., 2021). The combination of these three factors leads to limited availability and high cost of CGTs, making it challenging to reach commercial success while supporting broad and equitable patient access (Sainatham et al., 2024; Bashor et al., 2022).

To tackle this challenge, the authors of this article partnered in the PAT4CGT consortium that aims to develop a miniaturised process analytical technology (PAT) platform, tailored specifically for CGT manufacturing. Advanced PAT is critical for process understanding in R&D and is also becoming a key tool for process monitoring during clinical and commercial manufacturing (Clegg et al., 2020; Gargalo et al., 2020). This trend is aligned with the concept of Industry 4.0, that aims to transform manufacturing and production systems through the integration of advanced digital technologies (Arden et al., 2021). Adoption of Industry 4.0 principles in CGT is a paradigm shift, introducing cutting-edge, digital technologies, and moving away from centralised, manual and paper-led, towards distributed, automated and knowledge-driven CGT manufacturing processes (Elsallab and Maus, 2023). In our opinion, digital solutions and innovative approaches to PAT that support scalable production are essential to deliver on the promise of these novel treatment modalities. Thus, the goal of the PAT4CGT project is to lay the foundation for minimally-invasive monitoring of critical process parameters (CPPs) in CGT. To this end, we develop a standalone, closed, automated, and miniaturised sensor technology platform for at-line monitoring of CPPs, suited for applications in CGT manufacturing.

## 2 Closed, automated and single-use tools for cell culture and processing

**FIGURE 1:**
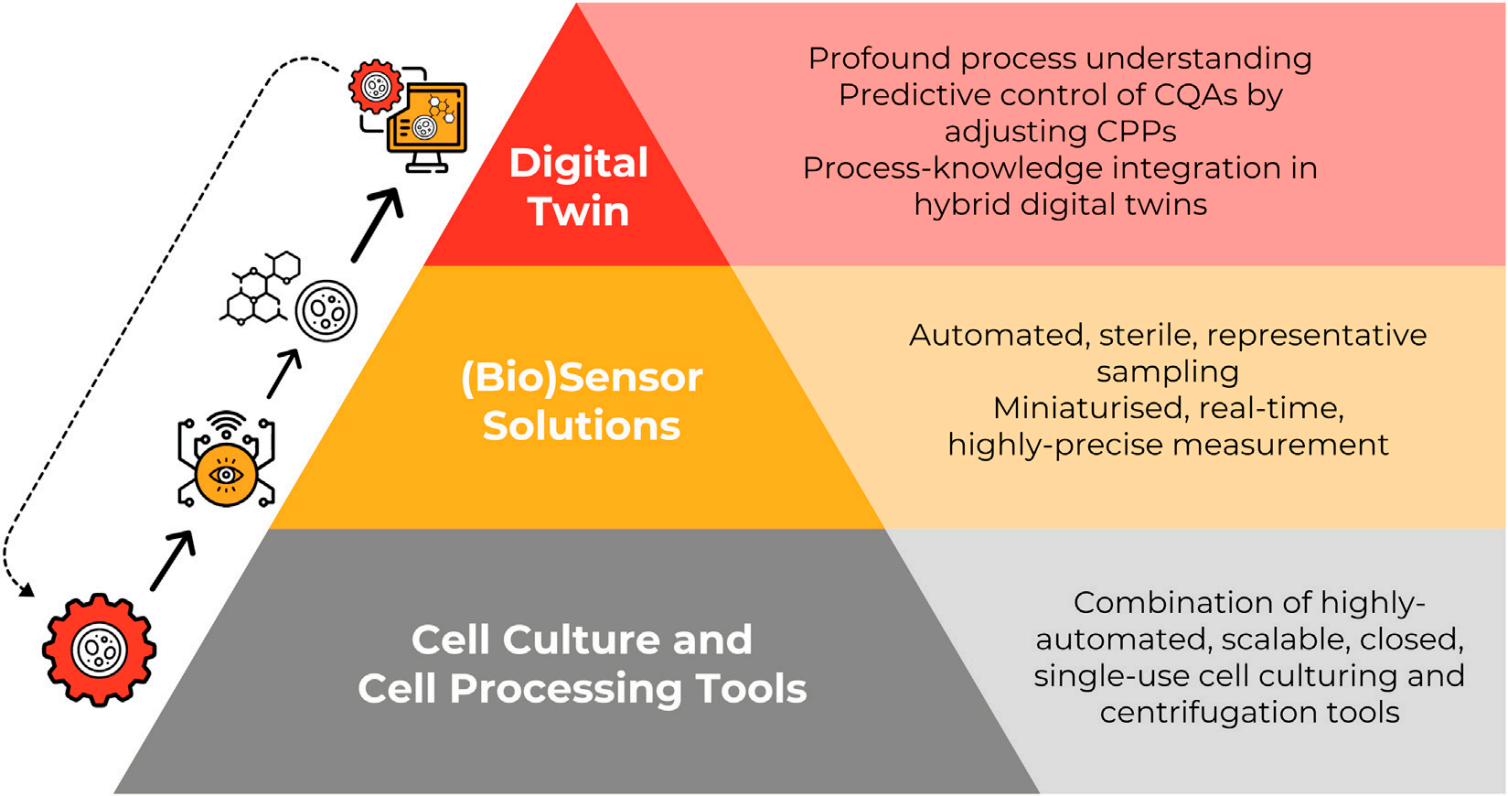
PAT4CGT’s hierarchy of CGT 4.0.

We believe that three layers of innovation are necessary to achieve the transformation towards CGT 4.0 (Figure 1). The first and fundamental layer consists of closed, automated and single-use tools that perform two unit operations at the core of all bioprocesses: cell culture (Mizukami et al., 2020) and cell processing (Li et al., 2021). These key steps are traditionally performed in bioreactors and centrifugation systems, respectively. Single-use and closed tools have become the standard in small- and mid-scale production of biologics and are increasingly used in large scale processes (Langer and Rader, 2014). While there are single-use technologies implemented in large-scale upstream processes, single-use centrifugation systems remain hard to scale. Overall, there is a critical need for novel approaches (Giorgioni et al., 2024) that take into consideration the unique properties that set CGT products apart from previous generations of biopharmaceutical products (Verbarendse et al., 2023; Doulgkeroglou et al., 2020).

In the case of autologous, gene-edited cell therapies, innovative solutions are needed to perform the complex, multi-step processes in a streamlined sequence of unit operations. Replacing manual operations with automation in closed systems (Moutsatsou et al., 2019; Melocchi et al., 2025) has the potential to increase process standardisation, robustness, scalability and reproducibility while lowering labour and clean room infrastructure costs (Lopes et al., 2018; McCoy et al., 2020; Nießing et al., 2021). There is empirical evidence (Francis et al., 2023) that process automation (Ahmadi et al., 2025) can substantially reduce the “hands-on” operator time required for their manufacturing of CAR T-cell therapies (Lock et al., 2022). Similarly, higher transduction and lower variability between batches were obtained for the production of a haemopoietic stem cell therapy using a closed, semi-automated approach, when compared to the standard manual process (Papanikolaou et al., 2019). Novel closed and automated solutions enabling end-to-end manufacturing of autologous cell therapies will be necessary to transform the field and enable point of care production. While modular approaches are being implemented, we believe bringing all unit operations into a single device, and thus removing transfer of cells between tools, will lead to higher process yields and product quality. Solutions supporting *ex vivo* cell manipulations across a range of volumes and cell numbers will also be instrumental in unlocking the full potential of these personalised treatments. Furthermore, scalability to a full patient dose within the same device has the potential to significantly reduce the costs and time currently invested in technology transfer during the transition from pre-clinical to commercial stage.

In the case of gene therapy, the cryopreservation and thawing of the master cell bank is a critical first step in the manufacturing process. Advanced methods that allow closed handling of cell banks use either the (high cell density) cell bag-based approach or a microfluidic-based system. Immortalised cell lines (e.g., HEK-derived cells) are already characterised and automated cell culture technologies exist for viral vector manufacturing (Song et al., 2024). However, the industry is still lacking fully automated workflows covering all steps, from cell expansion, virus production and purification up to the final formulation (Destro et al., 2024). The traditional tools currently used to perform these unit operations, such as flatware flask for cell culture or ultracentrifugation for virus purification, are unsatisfactory as they not only hinder scalability but also lack the possibility of continuous data acquisition.

## 3 Miniaturised sensors for process monitoring

The middle layer of innovation towards CGT 4.0 is composed of sensor solutions that can measure CPPs in real-time, providing vital information about the process status. Monitoring capabilities are critical to capture data into a record that is used in process development or required for batch documentation but also to provide the necessary inputs for process control (Herwig et al., 2020). The information about CPPs facilitates decision making through early detection of deviations and out of specification (OOS) events. This can enable timely intervention to improve the manufacturing success rate, as well as minimisation of losses associated with batch failure. To achieve this, a variety of offline, at-line and inline tools exist but their integration into CGT processes remains questionable (Gargalo et al., 2020). There are currently two concurring PAT approaches: (i) measuring a sample of the process with a traditionally offline, high-precision method (e.g., HPLC-MS) but automating the sampling process, or (ii) relying on an *in situ* method (e.g., spectroscopy) that often has an indirect measurement principle and requires extensive data analysis but can be applied in a closed system without the risk of contamination.

In our opinion, sensor arrays for the monitoring of traditional biologics’ production–often limited to temperature, pH, dissolved oxygen and CO_2_ – are insufficient to characterise the status of a complex CGT production batch because tracking of cell metabolism is required for process development (Nikita et al., 2023; Chen et al., 2024; Kimura et al., 2019; Román et al., 2018). While relying on historical process data to design a medium supplementation strategy, the absence of continuous–or at least frequent–monitoring of cell metabolism limits process flexibility to adjust media components when important changes occur, hindering productivity or product quality. Inline spectroscopic sensors, such as infrared, Raman and dielectric spectroscopy, offer capabilities to monitor, nutrients, metabolites (Christie et al., 2024; Costa et al., 2024; Marienberg et al., 2024; Abu-Absi et al., 2011) or cell count (Sripada et al., 2024; Bergin et al., 2022). However, these methods are influenced by non-specific (background) variations (Sripada et al., 2024). Moreover, data interpretation, validation and software integration into process control, as well as the physical integration of a probe into innovative, small-scale manufacturing systems of CGTs are challenging (Sripada et al., 2024).

The limitations due to background signals with the above-mentioned methods can be overcome by the use of a specific recognition element, such as an antibody-based or enzymatic biosensor (Fedi et al., 2022; Moser et al., 2002). These, however, are at-line methods that require a representative sample. The sampling technique must be tailored to the unique requirements of CGT production, mainly to single-use applications, across a wide range of process scales. Sterile sampling opens the possibility for further downstream processing and offline analytics. In general, the combination of automated, *in situ* collection of samples from a manufacturing platform in a closed, single-use environment with a highly precise, specific method has the potential to lower the risk of contamination associated with manual sampling and provide continuous/frequent measurements. This will play an important role in increasing the availability of data for subsequent analysis.

## 4 Digital twin for process knowledge integration and control

This leads us to the top layer of CGT 4.0, which is a data analytics solution allowing for the transformation of the information, collected by the analytical tools, into actionable knowledge, to efficiently manage the process lifecycle. In our opinion, a mathematical model of the manufacturing process, also known as a “digital twin”, is an essential tool for knowledge integration, with the aim of enabling predictive control of the critical quality attributes (CQAs) by adjusting the CPPs (Canzoneri et al., 2021). Historically, experimental data is used for mechanistic modelling approaches (e.g., material balance of substrates and metabolites) to describe microbial systems (Muloiwa et al., 2020). These methods have been successfully adapted to more complex cases, such as Chinese hamster ovary (CHO) cells (González-Hernández and Perré, 2024; Park et al., 2021). The mechanistic assumptions are not universal but the different modelling workflows can be transferred between cell types (Wang et al., 2024). However, their imminent translation to CGT processes is limited by the generally low process understanding of CGTs (Hort et al., 2022; Canova et al., 2023; Triantafyllou et al., 2024). Therefore, the prerequisite of developing accurate predictive models is to clearly define, then capture the time-resolved values of the CPPs and CQAs of CGT processes (Johanna et al., 2023), for which automated, *in situ* analytical solutions can play a central role.

In our view, purely data-driven, neural network methods could be used to develop models of CGT processes (Yatipanthalawa and Gras, 2024; Emerson et al., 2020). For the generation of the large amounts of data that are necessary to train such models, novel, automated cell culturing tools could resolve the bottleneck of current production capacities. Another potential solution to the limited availability of data is to apply more sophisticated approaches and algorithms, such as hybrid modelling. Hybrid modelling combines mechanistic representations with data-driven methods (Schweidtmann et al., 2024). For instance, the long-short term memory (Ramos et al., 2024) and physics informed approaches (Yang et al., 2024), as well as the reinforced learning-based methods (Mowbray et al., 2023). However, the missing connection between the variability in the starting material (e.g., variability in cell phenotype between patients) and the process outcome, in the case of autologous cell therapies, poses an extra challenge. While classical mechanistic models can potentially describe the effect of nutrients and metabolites on cell growth, completely novel mechanistic representations are needed to describe cytotoxicity and similar efficacy CQAs for other CGTs. All things considered, hybrid modelling will be the approach that, in our opinion, will eventually prevail in the field of CGT, as its ability to incorporate simpler process knowledge, unconventional process parameters and mechanistic descriptions of efficacy CQAs allows users to achieve the desired prescriptive process control, as a key element of CGT 4.0.

Naturally, as with every novel technique, an acceptable solution to validate these models, depending on the model’s importance for the process, has to be found (BioPhorum, 2021). Active discussions in the filed indicate that more time is needed to establish a universal validation approach for good manufacturing practice (GMP) compliance (O'Connor et al., 2024; European Medicines Agency EMA, 2024). However, in our view, the definition of suitable regulation is a technical question that can be addressed through collaboration of all parties concerned.

## 5 Conclusion

We conclude that innovation across three layers is key to enable robust and scalable manufacturing of CGT products: closed and automated tools, robust and frequent process measurements and data analytics will enable the development and execution of well-characterised and adaptive production methods. The integration of these three layers into cohesive systems requires a multidisciplinary approach combining a profound understanding of the underlying biology as well as engineering skills to identify relationships between CPPs and CQAs and compile all process knowledge in a digital twin. Considering the complexity of the problem at hand, we wish to highlight the important role of innovation management and collaboration between experts from very diverse fields. Product engineering and design, cell biology and bioprocesses, sensors development, data modelling and material sciences, as well as end users and regulators of the technologies all need to come together to guide the industry towards the adoption of meaningful Industry 4.0 concepts applicable to CGT products, in order to provide novel therapeutic options to a vast number of patients.
